# *STK11* (*LKB1*) mutations in metastatic NSCLC: Prognostic value in the real world

**DOI:** 10.1371/journal.pone.0238358

**Published:** 2020-09-03

**Authors:** Norah J. Shire, Alyssa B. Klein, Asieh Golozar, Jenna M. Collins, Kathy H. Fraeman, Beth L. Nordstrom, Robert McEwen, Todd Hembrough, Naiyer A. Rizvi

**Affiliations:** 1 AstraZeneca, Gaithersburg, MD, United States of America; 2 Evidera, Waltham, MA, United States of America; 3 AstraZeneca, Cambridge, United Kingdom; 4 Division of Hematology and Oncology, Columbia University Medical Center, New York, NY, United States of America; University of Crete, GREECE

## Abstract

**Background:**

Mutations in *STK11* (*STK11*m) and frequently co-occurring *KRAS* mutations (*KRAS*m/*STK11*m) are associated with poor survival in metastatic NSCLC (mNSCLC) immuno-oncology trials. There are limited data regarding the prognostic significance of these mutations in a real-world setting.

**Methods:**

This retrospective cohort study analyzed de-identified electronic medical records from the Flatiron Clinico-Genomic database to identify patients with mNSCLC who had initiated first-line immunotherapy (IO; alone or in combination) or chemotherapy under routine care between January 1, 2013 and June 30, 2017. The primary objectives were to assess the prevalence of *STK11*m and *KRAS*m/*STK11*m and to determine associations of these mutations with overall and progression-free survival (OS, PFS).

**Results:**

Of 2407 patients with mNSCLC, *STK11*m and *KRAS*m/*STK11*m were present in 13.6% and 6.5% of patients, respectively. Worse OS outcomes were observed in patients with *STK11*m versus *STK11*wt mNSCLC receiving IO (first-line, HR [95% CI], 1.4 [0.9–2.3; p = 0.1]; second-line [subset of first-line cohort], HR, 1.6 [1.3–2.0; p = 0.0002]) or chemotherapy (first-line, HR, 1.4 [1.2–1.6; p < 0.0001]); PFS outcomes showed similar trends. *KRAS*m/*STK11*m double mutations were associated with worse OS and PFS outcomes versus *KRAS*wt/*STK11*wt with IO and chemotherapy, similar to the single mutation (*STK11*m vs *STK11*wt) findings.

**Conclusions:**

This large observational genomic study among patients receiving routine care highlights the negative prognostic impact of *STK11*m in patients with mNSCLC treated with IO or chemotherapy. These results complement previous clinical trial data and provide further evidence in the real world of a patient population that would benefit from new treatment options.

## Introduction

Lung cancer is among the most commonly diagnosed cancers, accounting for 11.6% of newly diagnosed cancers globally and 18.4% of all cancer deaths [1]. Non-small cell lung cancer (NSCLC) represents approximately 85% of all lung cancers, with nearly 70% of patients with NSCLC presenting in advanced stages of disease [1–4].

Until recently, the standard first-line treatment option for metastatic NSCLC consisted of platinum-based doublet chemotherapy regimens, which were associated with poor survival outcomes [4, 5]. Immune checkpoint inhibitors targeting programmed cell death-1 (PD-1) or its ligand (PD-L1) as monotherapy or in combination with chemotherapy have transformed the treatment landscape for patients with metastatic NSCLC, especially those without oncogenic driver mutations [6–11]. The first anti-PD-1 agents were approved by the US Food and Drug Administration (FDA) for pretreated metastatic NSCLC in October 2015 and the first approval of anti-PD-1 for first-line metastatic NSCLC occurred in October 2016.

Despite PD-1/PD-L1 inhibitors becoming a standard of care for patients with metastatic NSCLC, there remains a significant patient population who either do not respond or do not derive long-term survival benefits from these therapies. Biomarkers identifying those more or less likely to derive treatment benefit, including from immunotherapy (IO) and chemotherapy, may help avoid unnecessary toxicity. PD-L1 expression on tumor cells has been used to guide treatment selection, and more recently tumor mutational burden (TMB) has shown potential as a predictive biomarker for IO benefit [12–15]. Mutations in individual genes and co-mutation patterns have also been linked to patient response to standard chemotherapy and/or IO in advanced NSCLC [16–24].

Mutations in the *STK11* (or liver kinase B1 [*LKB1]*) gene (*STK11*m), found in approximately 5–30% of NSCLC cases [21, 25, 26], have recently been identified as an important regulator of resistance to anti-PD-1/PD-L1 therapies [18, 19, 22]. STK11 is a serine-threonine kinase that is an important regulator of cellular metabolism and energy sensing, and functions by activating AMP kinase (AMPK) and AMPK-related family members [27–29]. Loss of STK11 increases serine utilization and synthesis of S-adenosyl methionine (SAM), a substrate for multiple epigenetic silencing enzymes including DNMT1 and EZH2, that may impact the expression of genes that affect immune recognition including the DNA sensor, Stimulator of Interferon Genes (STING) [27, 28, 30]. *STK11* mutations are associated with an “immune cold” tumor microenvironment characterized by low or no PD-L1, low T-cell densities, high levels of granulocyte colony stimulating factor and IL-8 family cytokines, high density of neutrophil-like cells, and production of myeloid cell-recruiting chemokines such as IL-6 [19, 31, 32].

*STK11*m may co-occur with mutations in *KRAS* (*KRAS*m), a common oncogenic driver in NSCLC [33, 34], and the presence of dual *STK11* and *KRAS* mutations has been associated with a trend towards poorer survival outcomes in NSCLC in response to chemotherapy and IO [20, 21, 23].

There are limited data regarding the prognostic significance of *STK11*m and *KRAS*m/*STK11*m outside of clinical trials. Data from real-world patients will help describe the prevalence of these mutations and their impact on clinical outcomes within current routine treatment practice. This retrospective analysis assessed the association between *STK11*m, *KRAS*m/*STK11*m and survival outcomes in patients with metastatic NSCLC receiving IO (alone or in combination) or chemotherapy from January 1, 2013 to June 30, 2017 in US community oncology clinics and academic cancer centers.

## Patients and methods

### Study design

This retrospective cohort study used real-world data from de-identified electronic medical records from the Flatiron Clinico-Genomic database (CGDB) to identify patients with metastatic NSCLC who had initiated first-line IO (alone or in combination) or chemotherapy under routine clinical practice between January 1, 2013 and June 30, 2017. The Flatiron CGDB contains processed longitudinal electronic medical records data from patients in Flatiron Health’s network of over 280 community and academic cancer centers within the United States of America, including patient demographics, deep diagnosis information (e.g. staging, histopathology, and biomarkers), treatment, and outcomes (e.g. mortality). The Flatiron data are linked with comprehensive genomic profiling results from Foundation Medicine’s FoundationCORE database [35]. The generation and validation of the Flatiron CGDB as well as methods for clinical data extraction, de-identification of patient data, linkage of the clinical and genomic data, and tumor genotyping (using the FoundationOne platform) have been described previously [35].

The Flatiron CGDB data are anonymized and the study data complied with US patient confidentiality requirements. As the study used only existing de-identified patient records, Institutional Review Board approval and patient informed consent were not required.

The study selection period (January 1, 2013–June 30, 2017) encompasses the dates when multiple anti-PD-1/PD-L1 products were approved by the US FDA for pretreated metastatic NSCLC (nivolumab and pembrolizumab in October 2015, and atezolizumab in October 2016) and a shorter window of time following the approval of anti-PD-1 in the first-line setting (pembrolizumab in October 2016). Patients treated with IO products approved after June 30, 2017 would not have been captured, with the exception of off-label use. Patients were followed longitudinally until death or their last visit prior to data cutoff. Demographic information, smoking history (ever smoker [patients with any history of smoking] or never smoker [patients with no history of smoking]), stage at initial diagnosis, sites of metastases, cancer treatment, medical history, disease characteristics (including NSCLC histology), and data on tumor evaluation (including progression of the disease and response to treatment), were considered as appropriate.

### Patients

The study cohort included patients who had confirmed metastatic (stage IV) NSCLC at diagnosis or who had progressed to metastatic disease from an earlier stage. Eligible patients had received at least one line of therapy for their disease, had initiated first-line IO or chemotherapy between January 1, 2013 and June 30, 2017, had genomic testing results available, and were age 18 years or older. Patients with a record of stage IV NSCLC or first-line therapy for metastatic disease were included. For those patients who progressed to stage IV from diagnosis at an earlier stage, the earlier of the two dates of secondary tumor diagnosis or start of first-line therapy for metastatic disease was considered the date of first diagnosis of metastatic NSCLC. To allow for sufficient follow-up for clinical outcomes, patients entered the cohort no later than 12 months prior to data cutoff (June 30, 2018). The index date was the start date of first-line therapy for metastatic disease. Up to 12 months of baseline data prior to the index date were used for examining patients’ medical histories. Because of the importance of smoking status as a prognostic factor in studies of NSCLC, patients lacking data on smoking status were excluded from this study.

### Study outcomes

The primary objectives of this study were to describe the prevalence of *STK11*m and *KRAS*m*/STK11*m in the study cohort and to assess the association of these mutations with overall survival (OS) and progression-free survival (PFS) by line of therapy (first-line cohort [includes all patients who received ≥1 line of therapy; full cohort] or second-line cohort [includes only patients who received ≥2 lines of therapy; subset of the first-line/full cohort]). Exploratory analyses included assessment of real-world response rate.

The *STK11* mutations were prioritized on the basis of their putative effect on protein function according to a previously described scheme [36]. The mutations thought to abrogate STK11 protein function included exon-level deletions, truncating structural rearrangements and point mutations leading to frameshifts, nonsense mutations, and splice site alterations.

Prevalence of *STK11*m or *KRAS*m was calculated as the percentage of patients with a positive result for each gene among all patients with complete data for the respective gene. Prevalence was summarized based on type of therapy (IO or chemotherapy) in the first- and second-line cohorts, and based on histology (squamous or non-squamous). The type of treatment received for metastatic disease during first-line therapy was assessed based on Flatiron-defined categories (IO agents, chemotherapy [platinum agents or other chemotherapy/targeted agents]).

OS for each patient was defined as the time to death from initiation of line of therapy (first- or second-line) and analyzed by therapy type (IO or chemotherapy) and mutation status. The month and year of death are noted in the Flatiron database and therefore the day of death was imputed as the maximum of the mid-point of the month of death or the last activity date across all medical records.

Real-world progression was determined by physician assessment as described previously [37]; the date and type of progression (actual progression [based on radiographic evidence, pathologic evidence, or clinical assessment], pseudo-progression, or mixed progression) were recorded in the Flatiron database and comprised variables within the dataset. PFS was defined as the time until the earliest record of actual disease progression or death from any cause from initiation of line of therapy (first- or second-line). PFS was analyzed by therapy type (IO or chemotherapy) and mutation status.

Response rates were investigated by calculating the numbers and percentages of patients with a response (Flatiron includes a field for maximum response [coded as complete, partial, or no response] for each line of treatment where response was assessed) among patients with known *STK11* status, as described previously [38]. However, response data are not often recorded as part of the patients’ medical records (thus, these data are not available for many patients) and the assessment of response data in Flatiron is not currently validated.

### Statistical analysis

Statistical analyses were conducted using SAS Enterprise Guide 7.1 (Cary, NC, USA), and statistical tests were two-sided with a significance level of 0.05.

Demographic and clinical characteristics were summarized, both for the cohort overall and separately by line of therapy and by therapy type (IO vs chemotherapy), with the median, and range for continuous variables and n (%) for categorical variables. Median OS and PFS were calculated along with 95% confidence intervals (CIs); censoring occurred at the last activity date for those patients without a defined clinical outcome (death or progression).

The associations between mutation status and OS, PFS, and real-world response rates were examined through descriptive analyses, including Kaplan-Meier plots of OS and PFS. Cox proportional hazards models were used for between-group comparisons of OS and PFS. The Cox models were fitted with all baseline characteristics as covariates (including age at start of the treatment line, sex, race, smoking status, stage of cancer at initial diagnosis, performance status at [or within 60 days before] start of the treatment line, Charlson Comorbidity Index at start of treatment line, *ALK* status, and *KRAS* status) and forward selection was used to eliminate non-significant variables, using p < 0.1 as the criterion for retaining variables in the final model.

## Results

### Patient characteristics

A total of 5250 patients with documented NSCLC from the Flatiron network with linked data in the Flatiron CGDB were available for analysis. Of these, 2407 patients who received first-line therapy for metastatic disease between January 1, 2013 and June 30, 2017 were included in the final study cohort, based on the inclusion/exclusion criteria described in the “Patients and methods” section. The sample selection process is shown in S1 Fig in S1 File.

In the overall metastatic NSCLC treatment cohort (2407 patients), a total of 2137 (88.8%) patients received chemotherapy during first-line therapy, of which 1580 (65.6%) received platinum-based chemotherapy (cisplatin or carboplatin). A total of 270 (11.2%) patients received IO agents (ipilimumab, nivolumab, pembrolizumab, or atezolizumab) alone or in combination during first-line therapy.

Patient characteristics for the study cohort are shown in Table 1. In the overall population (all patients who received first-line therapy), the median age of patients was 67 years (range 27–84), almost half (48.9%) were male, 73.6% were white, and 77.9% had non-squamous histology. Eastern Cooperative Oncology Group performance status (ECOG PS) data were available for 64.7% of patients. A total of 21.9% and 33.8% of all patients had ECOG PS 0 and 1, respectively; 9% had PS >1. *EGFR* mutation was found in 9.1% of the study population, although half of all patients had unknown *EGFR* status (as these data were not available for all patients in the Flatiron Health records). Among the 2407 patients in the study cohort who received first-line therapy, 1533 patients also received second-line treatment with IO or chemotherapy. Patient characteristics analyzed at the start of each line of therapy and type of therapy were generally consistent with the overall patient population (Table 1). A total of 270 patients received IO in the first-line and 670 in the second-line; 1493 patients in the study cohort never received IO (in either the first-line or second-line setting). Among 863 patients receiving second-line chemotherapy, 87 (10.1%) had received IO in the first-line setting.

**Table 1:** Patient characteristics at start of line of therapy.

		First-line cohort			Second-line cohort		
		All	IO	CT	All	IO	CT
		(n = 2407)	(n = 270)	(n = 2137)	(n = 1533)	(n = 670)	(n = 863)
Median age (range), years		67 (27‒84)	69 (35‒84)	67 (27‒84)	67 (27‒85)	69 (27‒85)	66 (33‒85)
Sex, n (%)	Male	1178 (48.9)	145 (53.7)	1033 (48.3)	737 (48.1)	315 (47.0)	422 (48.9)
	Female	1229 (51.1)	125 (46.3)	1104 (51.7)	796 (51.9)	355 (53.0)	441 (51.1)
Race, n (%)	White	1771 (73.6)	207 (76.7)	1564 (73.3)	1136 (74.1)	507 (75.7)	629 (72.9)
	Black/African American	164 (6.8)	21 (7.8)	143 (6.7)	110 (7.2)	54 (8.1)	56 (6.5)
	Asian	73 (3.0)	2 (0.7)	71 (3.3)	58 (3.8)	13 (1.9)	45 (5.2)
	Other	201 (8.4)	17 (6.3)	184 (8.6)	122 (8.0)	48 (7.2)	74 (8.6)
	Unknown	196 (8.1)	23 (8.5)	173 (8.1)	107 (7.0)	48 (7.2)	59 (6.8)
ECOG PS ≤60 days before line of therapy,^a^ n (%)	0	526 (21.9)	55 (20.4)	471 (22.0)	289 (18.9)	121 (18.1)	168 (19.5)
	1	813 (33.8)	104 (38.5)	709 (33.2)	655 (42.7)	314 (46.9)	341 (39.5)
	2	171 (7.1)	25 (9.3)	146 (6.8)	193 (12.6)	105 (15.7)	88 (10.2)
	3	46 (1.9)	8 (3.0)	38 (1.8)	37 (2.4)	22 (3.3)	15 (1.7)
	4	1 (0.04)	0 (0.0)	1 (0.05)	1 (0.1)	1 (0.1)	0 (0.0)
	Not documented	850 (35.3)	78 (28.9)	772 (36.1)	358 (23.4)	107 (16.0)	251 (29.1)
*EGFR* alterations	Positive	218 (9.1)	3 (1.1)	215 (10.1)	170 (11.1)	29 (4.3)	141 (16.3)
	Negative	985 (40.9)	127 (47.0)	858 (40.1)	680 (44.4)	307 (45.8)	373 (43.2)
	Unknown status	1204 (50.0)	140 (51.9)	1064 (49.8)	683 (44.5)	334 (49.9)	349 (40.4)
*ALK* rearrangement	Positive	39 (1.6)	1 (0.4)	38 (1.8)	29 (1.9)	3 (0.4)	26 (3.0)
	Negative	1052 (43.7)	111 (41.1)	941 (44.0)	753 (49.1)	310 (46.3)	443 (51.3)
	Unknown status	1316 (54.7)	158 (58.5)	1158 (54.2)	751 (49.0)	357 (53.3)	394 (45.7)
History of smoking, n (%)	Yes	1922 (79.9)	235 (87.0)	1687 (78.9)	1200 (78.3)	567 (84.6)	633 (73.3)
	No	485 (20.1)	35 (13.0)	450 (21.1)	333 (21.7)	103 (15.4)	230 (26.7)
Histology, n (%)	Non-squamous	1874 (77.9)	187 (69.3)	1687 (78.9)	1181 (77.0)	498 (74.3)	683 (79.1)

^a^ 0, fully active; 1, restricted in physically strenuous activity but ambulatory, able to carry out light work; 2, ambulatory and capable of self-care but unable to carry out any work activities; 3, capable of only limited self-care; 4, completely disabled. CT: chemotherapy; ECOG PS: Eastern Cooperative Oncology Group performance status; IO: immunotherapy.

A total of 77.9% of patients had non-squamous histology; patient characteristics in the non-squamous subgroup were consistent with the overall population shown in Table 1.

### Prevalence of *STK11*m and *KRAS*m*/STK11*m

In the 2407 patients included in the study cohort, the prevalence of *STK11*m was 13.6% (Table 2). *KRAS*m/*STK11*m was found in 6.5% of all patients. Among patients with *STK11*m, almost half (47.9%) also harbored *KRAS*m. In patients with non-squamous metastatic NSCLC (n = 1874), the prevalence of *STK11*m was 15.7% and the prevalence of *KRAS*m/*STK11*m was 8.0% (Table 2). In patients with squamous metastatic NSCLC (n = 441), the prevalence of *STK11*m was 5.0%.

**Table 2:** *STK11* and *KRAS*/*STK11* mutation prevalence in all patients with metastatic NSCLC and patients with non-squamous metastatic NSCLC.

Patients, n (%)	All patients (n = 2407)	Non-squamous histology (n = 1874)
*STK11m*	328 (13.6)	295 (15.7)
*KRAS*m	734 (30.5)	660 (35.2)
*KRAS*m/*STK11*wt	577 (24.0)	511 (27.3)
*KRAS*m/*STK11*m	157 (6.5)	149 (8.0)
*KRAS*wt/*STK11*m	171 (7.1)	146 (7.8)
*KRAS*wt/*STK11*wt	1502 (62.4)	1068 (57.0)

m: mutant; NSCLC: non-small cell lung cancer; wt: wild type.

Prevalence of the mutations in the first- and second-line cohorts based on the type of therapy is summarized in S1 Table in S1 File. The prevalence of *STK11*m was 13.6% in the first-line cohort (i.e. all patients) and 12.7% in the second-line cohort. The prevalence of *STK11*m was 13.5% in patients who received first-line chemotherapy and 9.6% in those who received second-line chemotherapy; the prevalence was 14.8% and 16.6% in in patients receiving first-line and second-line IO, respectively.

### Survival outcomes

**Fig 1:**
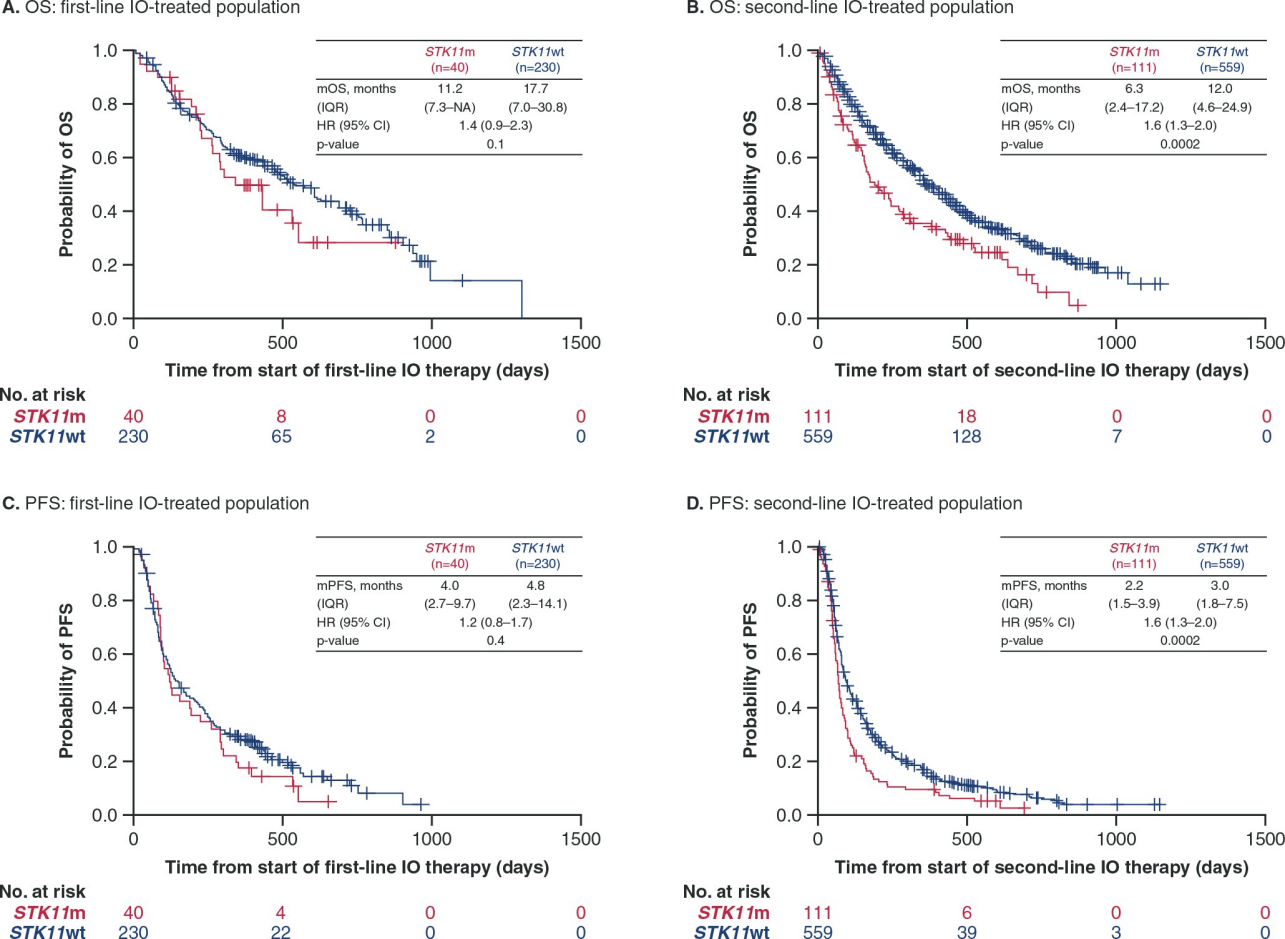
Overall survival and progression-free survival from start of first-line and second-line IO by *STK11* mutation status. OS and PFS were assessed as time to event from initiation of line of therapy (first-line or second-line). HR values reported are adjusted HRs calculated using multivariate Cox regression models (variables in the final adjusted multivariate analysis in the first-line IO group included race, ECOG status ≤60 days before start of line 1, and stage at initial diagnosis for OS [panel **A**] and *ALK* status for PFS [panel **C**]; for the second-line IO group, ECOG status ≤60 days before start of line 2 was included for OS and PFS [panels **B** and **D**]). CI: confidence interval; ECOG: Eastern Cooperative Oncology Group; HR: hazard ratio; IO: immunotherapy; IQR: interquartile range; OS: overall survival; PFS: progression-free survival.

**Fig 2:**
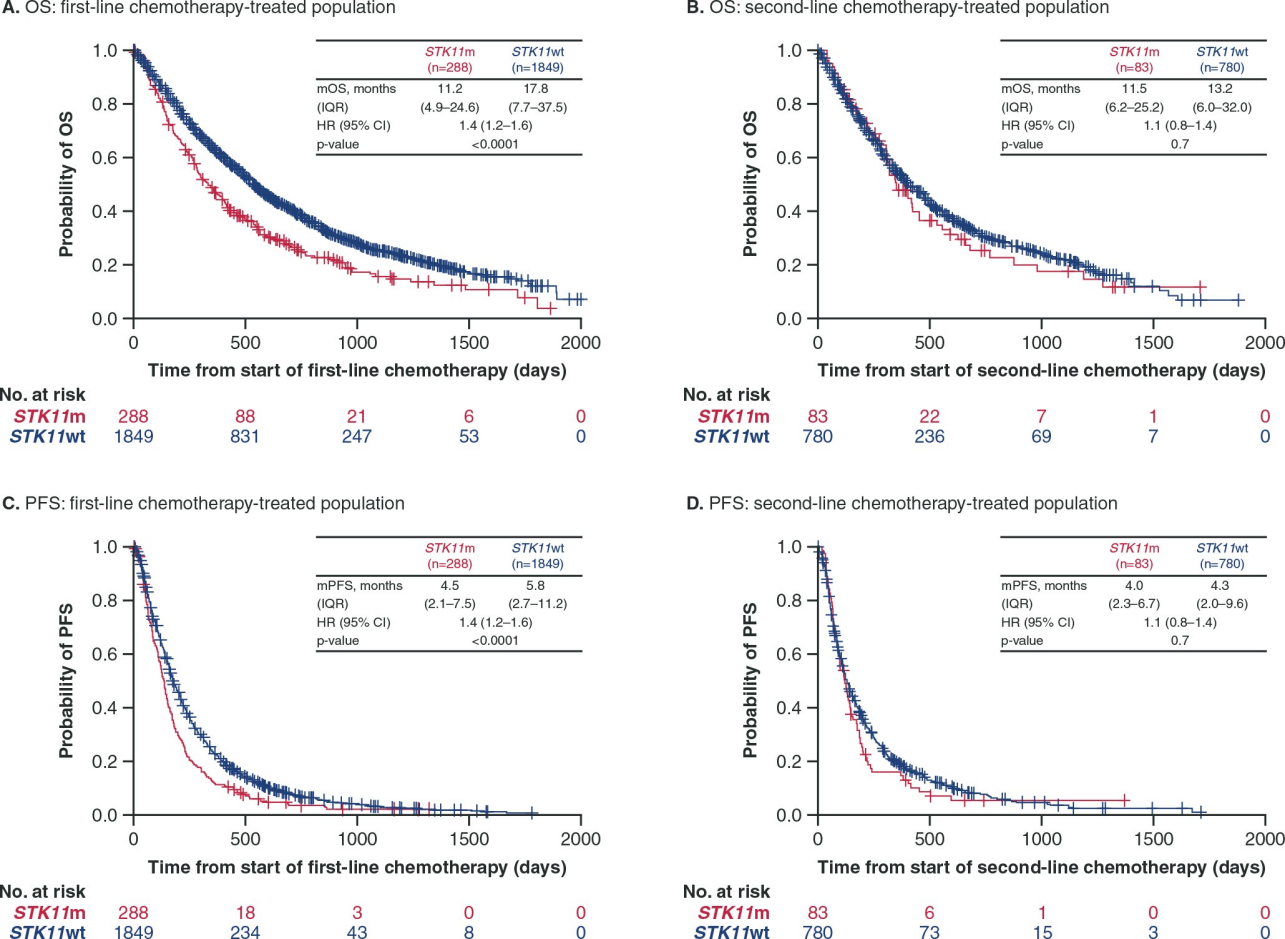
Overall survival and progression-free survival from start of first-line and second-line chemotherapy by *STK11* mutation status. OS and PFS were assessed as time to event from initiation of line of therapy (first-line or second-line). HR values reported are adjusted HRs calculated using multivariate Cox regression models (variables in the final adjusted multivariate analysis in the first-line chemotherapy group included age at start of line 1, race, gender, smoking history, ECOG status ≤60 days before start of line 1, and stage at initial diagnosis for OS and PFS [panels **A** and **C**]; for the second-line chemotherapy group, race, smoking history, and ECOG status ≤60 days before start of line 2 were included for OS and PFS [panels **B** and **D**]). CI: confidence interval; ECOG: Eastern Cooperative Oncology Group; HR: hazard ratio; mOS: median overall survival; mPFS: median progression-free survival; OS: overall survival; PFS: progression-free survival.

OS and PFS outcomes were inferior in patients with *STK11*m compared with patients with *STK11*wt treated with IO or chemotherapy. In the first-line IO group, median OS was numerically shorter for patients with *STK11*m compared with patients with *STK11*wt (11.2 vs 17.7 months; HR, 1.4 [95% CI, 0.9–2.3]) (Fig 1A). The difference in median OS was more pronounced in the second-line IO group for patients with *STK11*m versus *STK11*wt (6.3 vs 12.0 months; HR, 1.6 [95% CI, 1.3–2.0]) (Fig 1B). The PFS results showed similar trends to those seen with OS (Fig 1C and 1D); median PFS in patients with *STK11*m versus *STK11*wt was 4.0 versus 4.8 months (HR, 1.2 [95% CI, 0.8–1.7]) in the first-line IO group and 2.2 versus 3.0 (HR, 1.6 [95% CI, 1.3–2.0]) in the second-line IO group. In the first-line chemotherapy group, median OS was shorter in patients with *STK11*m versus *STK11*wt (11.2 vs 17.8 months; HR, 1.4 [95% CI, 1.2–1.6]) (Fig 2A). Median OS in the second-line chemotherapy group for patients with *STK11*m versus *STK11*wt was 11.5 months versus 13.2 months (HR, 1.1 [95% CI, 0.8–1.4]) (Fig 2B). Similar trends were seen with PFS; in patients receiving first-line chemotherapy, *STK11*m was associated with a shorter median PFS compared with *STK11*wt (4.5 vs 5.8 months, HR, 1.4 [95% CI, 1.2–1.6]) (Fig 2C). In the second-line chemotherapy group, median PFS was 4.0 versus 4.3 months in patients with *STK11*m versus *STK11*wt (HR, 1.1 [95% CI, 0.8–1.4]) (Fig 2D).

In patients with non-squamous NSCLC, outcomes were generally consistent with the overall population (Table 3).

**Table 3:** Overall survival and progression-free survival from start of first-line and second-line IO and chemotherapy by *STK11* mutation status (patients with non-squamous NSCLC).

	Immunotherapy		Chemotherapy	
	First-line therapy (n = 187)	Second-line therapy (n = 498)	First-line therapy (n = 1687)	Second-line therapy (n = 683)
Median OS, months (IQR)				
*STK11*m	14.2 (7.3‒NA)	6.6 (2.4‒20.1)	11.7 (5.1‒25.2)	13.1 (7.2‒25.2)
*STK11*wt	20.1 (7.4‒42.8)	13.6 (4.8‒27.9)	18.9 (8.0‒41.0)	15.2 (6.5‒38.0)
HR (95% CI)	1.4 (0.8‒2.3)^a^	1.7 (1.3‒2.2)^b^^,^^h^	1.4 (1.2‒1.7)^c^^,^^h^	1.1 (0.8‒1.4)^d^
Median PFS, months (IQR)				
*STK11*m	4.1 (2.7‒9.8)	2.2 (1.5‒3.7)	4.5 (2.1‒7.5)	4.2 (2.3‒6.7)
*STK11*wt	5.4 (2.4‒17.0)	3.1 (1.8‒7.7)	6.1 (2.8‒11.9)	4.5 (2.1‒10.1)
HR (95% CI)	1.4 (0.9‒2.0)^e^	1.6 (1.2‒2.0)^d^^,^^h^	1.4 (1.2‒1.6)^f^^,^^h^	1.1 (0.8‒1.4)^g^

OS and PFS were assessed as time to event from initiation of line of therapy (first-line or second-line). All HR values reported are adjusted HRs calculated using multivariate Cox regression models. Variables in the final adjusted multivariate analysis

^a^ Race, stage at initial diagnosis, and ECOG status ≤60 days before start of line 1.

^b^ ECOG status ≤60 days before start of line 2.

^c^ Race, gender, smoking history, stage at initial diagnosis, and ECOG status ≤60 days before start of line 1.

^d^ Smoking history and ECOG status ≤60 days before start of line 2.

^e^ Race, smoking history, ECOG status ≤60 days before start of line 1, and *ALK* status.

^f^ Gender, smoking history, stage at initial diagnosis, and ECOG status ≤60 days before start of line 1.

^g^ Gender, smoking history, and stage at initial diagnosis.

^h^ p-value <0.001. CI: confidence interval; ECOG: Eastern Cooperative Oncology Group; HR: hazard ratio; IO: immunotherapy; IQR: interquartile range; m: mutant; NSCLC: non-small cell lung cancer; OS: overall survival; PFS: progression-free survival; wt: wild type.

Similar to the results with *STK11*m versus *STK11*wt, patients with co-mutations in *KRAS* (*KRAS*m/*STK11*m) treated with IO or chemotherapy had worse OS and PFS outcomes compared with their wild-type counterparts (*KRAS*wt/*STK11*wt) (Table 4). Median OS was numerically shorter for patients with KRAS*m*/*STK11*m than for those with *KRAS*wt/*STK11*wt in the first-line IO group (10.0 vs 16.3 months; HR, 1.5 [95% CI, 0.7–2.9]); this difference was more pronounced in the second-line IO group (6.9 vs 12.0 months; HR, 1.6 [95% CI, 1.2–2.3]). Similarly, *KRAS*m/*STK11*m was associated with shorter median OS compared with *KRAS*wt/*STK11*wt in the first-line chemotherapy group (11.7 vs 18.2 months; HR, 1.6 [95% CI, 1.3–1.9]). In the second-line chemotherapy group, the median OS was 11.3 months for *KRAS*m/*STK11*m and 13.2 months for *KRAS*wt/*STK11*wt (HR, 1.3 [95% CI, 0.9–1.8]). PFS results showed similar trends to the OS results for both IO and chemotherapy groups, although most differences were not statistically significant, possibly due to the small sample sizes.

**Table 4:** Overall survival and progression-free survival from start of first-line and second-line IO and chemotherapy by *KRAS/STK11* mutation status (all patients).

	Immunotherapy		Chemotherapy	
	First-line therapy (n = 166)	Second-line therapy (n = 427)	First-line therapy (n = 1493)	Second-line therapy (n = 650)
Median OS, months (IQR)				
*KRAS*m/*STK11*m	10.0 (7.3‒NA)	6.9 (2.4‒21.9)	11.7 (5.2‒23.5)	11.3 (7.2‒25.2)
*KRAS*wt/*STK11*wt	16.3 (6.0‒29.6)	12.0 (4.3‒26.9)	18.2 (8.1‒38.4)	13.2 (6.3‒33.5)
HR (95% CI)	1.5 (0.7‒2.9)^a^	1.6 (1.2‒2.3)^b^^,^^c^	1.6 (1.3‒1.9)^a^^,^^c^	1.3 (0.9‒1.8)^b^
Median PFS, months (IQR)				
*KRAS*m/*STK11*m	4.1 (2.5‒9.6)	2.2 (1.5‒3.0)	4.5 (2.1‒7.5)	4.4 (2.8‒6.7)
*KRAS*wt/*STK11*wt	4.4 (2.1‒11.7)	2.8 (1.7‒7.0)	5.9 (2.8‒11.3)	4.3 (2.2‒9.7)
HR (95% CI)	1.3 (0.8‒2.2)^a^	1.8 (1.4‒2.4)^b^^,^^c^	1.4 (1.2‒1.7)^a^^,^^c^	1.1 (0.8‒1.5)^b^

OS and PFS were assessed as time to event from initiation of line of therapy (first-line or second-line). All HR values reported are adjusted HRs calculated using multivariate Cox regression models; the models included all patients, including those with *KRAS*m/*STK11*wt and *KRAS*wt/*STK11*m (total n = 270 [first-line immunotherapy]; 670 [second-line immunotherapy]; 2137 [first-line chemotherapy]; and 863 [second-line chemotherapy]). Variables in the final adjusted multivariate analysis

^a^ Age at start of line 1, gender, smoking history, ECOG status ≤60 days before start of line 1, and stage at initial diagnosis.

^b^ Age at start of line 2, gender, smoking history, ECOG status ≤60 days before start of line 2, stage at initial diagnosis, and first-line treatment type.

^c^ p-value <0.005. CI: confidence interval; CT: chemotherapy; ECOG: Eastern Cooperative Oncology Group; HR: hazard ratio; IO: immunotherapy; IQR: interquartile range; m: mutant; OS: overall survival; PFS: progression-free survival; wt: wild type.

In patients with non-squamous NSCLC and co-mutations in *KRAS* and *STK11*, outcomes were generally consistent with the overall population.

### Response rate

In both the IO and chemotherapy treatment groups, mean response rates were slightly lower for patients with *STK11m* compared with *STK11wt*, although the 95% CIs overlapped, reflecting the lack of available response data (S2 Table in S1 File).

## Discussion

This real-world study represents one of the largest cohorts of treated patients with metastatic NSCLC used to evaluate the correlation between *STK11*m and survival within the context of routine treatment practice. The prevalence of *STK11m* in this real-world study cohort for the overall population (13.6%) was within the range of previously reported findings in other retrospective observational studies and clinical trials [20, 21, 24–26, 33, 34]. Almost half of the patients with *STK11*m had a co-mutation in *KRAS*m, which is largely consistent with the co-mutation frequencies reported previously [20, 21, 24, 33].

In the current study, the survival outcomes analyzed by line and type of therapy suggest worse OS and PFS with *STK11*m versus *STK11*wt in the IO treatment group. The between-group differences were more pronounced in the patients receiving second-line IO compared with first-line IO, possibly because of the larger sample size (the number of patients in the first-line IO group was smaller, given the time period of the analysis [January 2013–June 2017] and the approval/availability of the IO products in the first-line setting [pembrolizumab was the first anti-PD-(L)1 approved in first-line metastatic NSCLC in October 2016]). The OS and PFS results in the first-line chemotherapy group were consistent with findings in the first- and second-line IO groups. In the second-line chemotherapy group, the OS and PFS results were similar between the *STK11*m and *STK11*wt patients. Interpretation of the survival outcomes data in the second-line chemotherapy-treated patients is complicated by the fact that some of these patients will have received IO in the first-line setting and others will have received first-line chemotherapy. However, further subdivisions of the second-line chemotherapy group according to first-line treatment type are not possible as this would lead to very small sample sizes.

Survival outcomes in the subset of patients with non-squamous histology (representing 78% of the study cohort) were consistent with the overall population.

The current data, in a large observational genomic study among patients receiving routine clinical care, support the association of *STK11*m with poor treatment outcomes previously observed in smaller clinical trial cohorts [18–24]. Shorter OS and reduced response rates have been observed in patients with *STK11*m versus *STK11*wt non-squamous metastatic NSCLC treated with durvalumab (with or without tremelimumab) across multiple phase 1/2 trials [18]. Similarly, in exploratory analysis from the phase 3 MYSTIC trial, across treatment arms patients with *STK11*m metastatic NSCLC had shorter median OS compared to patients with *STK11*wt metastatic NSCLC [24]. *STK11m* have been associated with inferior clinical outcomes with PD-1 blockade in multiple independent cohorts of *KRAS*m NSCLC, including patients with *KRAS*m non-squamous NSCLC treated with nivolumab in the CheckMate 057 study [19]. The authors of the study conclude that the effect likely extends to the entire non-squamous NSCLC population, regardless of *KRAS* status [19]. Similarly, in a retrospective, multicenter, international study, poor clinical outcomes were reported in patients with *STK11*m versus *STK11*wt non-squamous metastatic NSCLC treated with pembrolizumab plus platinum-based chemotherapy [22].

Our observations in patients with *KRAS*m/*STK11*m were similar to those in patients with *STK11*m only, suggesting no additional deterioration of outcomes in the double mutants. In some retrospective studies, patients with co-mutations in *KRAS* and *STK11* have shown a trend towards slightly poorer survival compared to patients with mutations in the individual genes (*KRAS*m only or *STK11*m only), although these analyses were not conclusive [19–21, 23].

The prognostic role of *STK11*m in combination with mutations in other genes (e.g. *KEAP1*) and based on PD-L1 expression and TMB is being evaluated in various clinical trials. Future analyses in the real-world setting with these additional parameters could yield valuable information to guide treatment selection.

There are several limitations to this study. In general, replicating a clinical trial population in a real-world setting is difficult due to multiple factors, including the extent of missing clinical information that is not routinely recorded by clinicians, variations in the reporting, selection biases arising from the use of diagnostic and therapeutic codes, incomplete or biased data on treatment responses, and under-reporting of comorbidities and of treatment received outside the oncology clinic setting, which may result in misclassification of treatments and outcomes, as has been described previously [35, 39]. The response results from database derived populations as studied here should be interpreted with caution as response data are not often recorded as part of the patients’ medical records (thus, these data are not available for many patients) and the assessment of response data in Flatiron is currently not validated (the response data obtained from patient medical records are different from assessment in clinical trials using standardized criteria such as RECIST [38]).

Another limitation is that the study was conducted over a time period in which the treatment landscape was rapidly evolving, with the introduction of immune checkpoint inhibitors and PD-L1 testing occurring part way through the study. In addition, the study selection period ended in June 2017 to permit appropriate follow-up for survival analyses. As such, certain elements of the results should to be considered within this context. First-line IO was approved by the US FDA towards the end of the study selection period (October 2016) and therefore there were more patients in the second-line IO group than in the first-line IO group. The first US FDA approval of IO plus chemotherapy was just before the end of the study selection period (May 2017) and therefore few patients treated with pembrolizumab plus platinum and other immunotherapy combinations approved after this date would have been captured during this study. Analysis of the data according to PD-L1 expression was not possible as the majority of the patients were not tested for PD-L1 during this time period.

The possibility of selection bias also needs to be considered while interpreting these results; this study population, which only included patients with clinico-genomic data who were treated at centers in the US, may not represent the entire metastatic NSCLC patient population [35]. However, in the validation study using the Flatiron CGDB, analyses of the NSCLC cohort consisting of 4064 patients replicated previously described clinico-genomic correlations, including the distribution of mutated genes (similar to previous descriptions from The Cancer Genome Atlas), association between driver mutations and clinical characteristics, and responses to targeted therapy, thus demonstrating the applicability of this method [35].

In conclusion, this large-scale real-world study highlights the prognostic value of *STK11*m in patients with metastatic NSCLC, whether treated with IO or with chemotherapy. The results were broadly consistent across different lines of therapy and histology. Further investigation into the relationship between *STK11*m and outcomes in patients treated with IO and/or chemotherapy is warranted in additional studies where the limitations associated with the current analysis period can be addressed.

## Supporting information

S1 File (DOCX)
