# Supplementary material for: STK11 (LKB1) mutations in metastatic NSCLC: Prognostic value in the real world
Source: PLoS One. 2020 Sep 3;15(9):e0238358. doi: 10.1371/journal.pone.0238358 (PMC7470384; doi:10.1371/journal.pone.0238358)
Supplement: S1 File — (DOCX) [file pone.0238358.s001.docx]

**Supporting Information**

**Table S1.** *STK11* and *KRAS*/*STK11* mutation prevalence in patients with metastatic NSCLC in the first- and second-line cohort based on type of therapy.

|  | **First-line cohort** | | | **Second-line cohort** | | |
| --- | --- | --- | --- | --- | --- | --- |
| **Patients, n (%)** | **Immunotherapy**  **(n = 270)** | **Chemotherapy**  **(n = 2137)** | **All patients  (n = 2407)** | **Immunotherapy**  **(n = 670)** | **Chemotherapy**  **(n = 863)** | **All patients  (n = 1533)** |
| *STK11m* | 40 (14.8) | 288 (13.5) | 328 (13.6) | 111 (16.6) | 83 (9.6) | 194 (12.7) |
| *KRAS*m | 98 (36.3) | 636 (29.8) | 734 (30.5) | 244 (36.4) | 214 (24.8) | 458 (29.9) |
| *KRAS*m/*STK11*wt | 81 (30) | 496 (23.2) | 577 (24.0) | 188 (28.1) | 172 (19.9) | 360 (23.5) |
| *KRAS*m/*STK11*m | 17 (6.3) | 140 (6.6) | 157 (6.5) | 56 (8.4) | 42 (4.9) | 98 (6.4) |
| *KRAS*wt/*STK11*m | 23 (8.5) | 148 (6.9) | 171 (7.1) | 55 (8.2) | 41 (4.8) | 96 (6.3) |
| *KRAS*wt/*STK11*wt | 149 (55.2) | 1353 (63.3) | 1502 (62.4) | 371 (55.4) | 608 (70.5) | 979 (63.9) |

m: mutant; NSCLC: non-small cell lung cancer; wt: wild type.

**Table S2.** Response rate for first-line and second-line IO and chemotherapy by *STK11* mutation status in all patients with known *STK11* status and known overall response.

|  | **Immunotherapy** | | **Chemotherapy** | |
| --- | --- | --- | --- | --- |
| **Response rate** | **First-line therapy**  **(n = 234)** | **Second-line therapy**  **(n = 493)** | **First-line therapy**  **(n = 698)** | **Second-line therapy**  **(n = 315)** |
| **STK11*m*** |  |  |  |  |
| n/N  % (95% CI) | 14/34  41.2 (24.6‒57.7) | 18/73  24.7 (14.8‒34.5) | 51/86  59.3 (48.9‒69.7) | 7/21  33.3 (13.2‒53.5) |
| **STK11*wt***  n/N  % (95% CI) | 89/200  44.5 (37.6­‒51.4) | 143/420  34.0 (29.5‒38.6) | 402/612  65.7 (61.9‒69.4) | 115/294  39.1 (33.5‒44.7) |

CI: confidence interval; m: mutant; wt: wild type.

**Fig S1.** Patient selection flow chart.


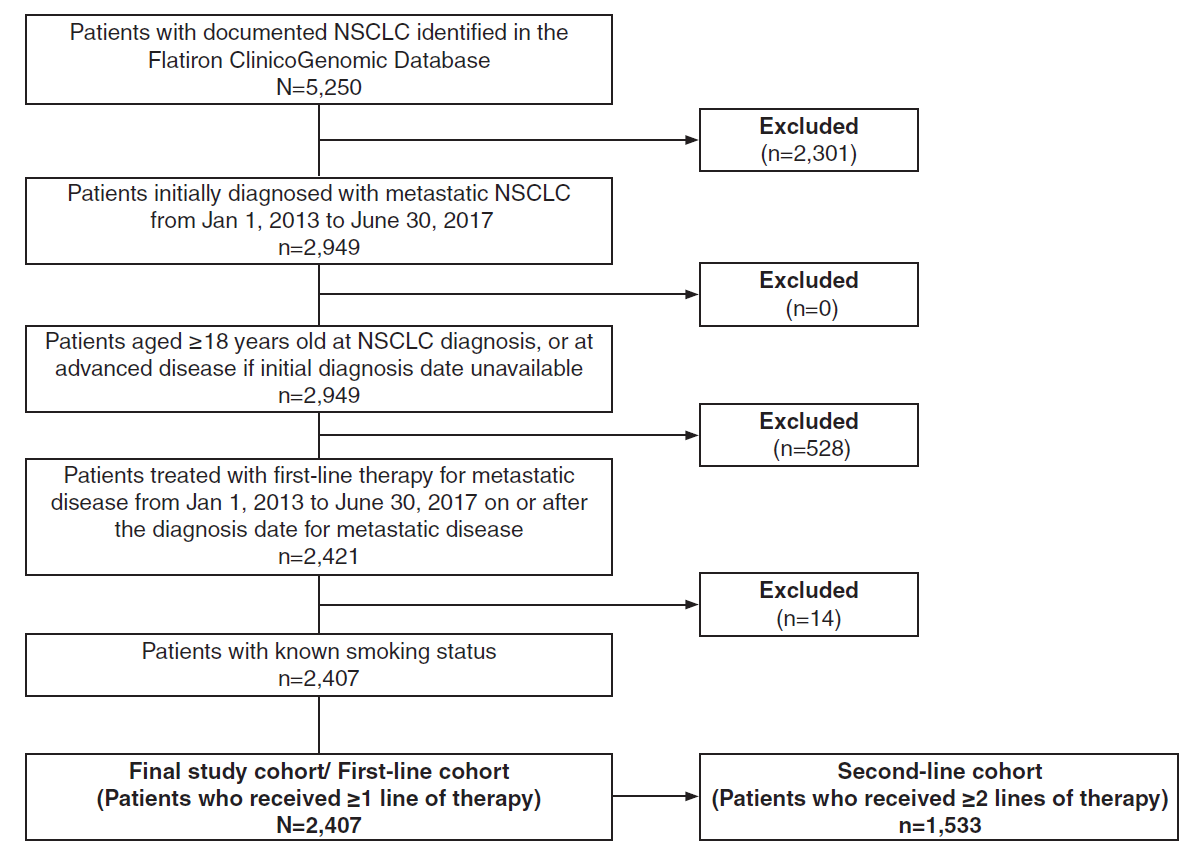


NSCLC: non-small cell lung cancer.
